# Cervical spine injuries occurring at the beach: epidemiology, mechanism of injury and risk factors

**DOI:** 10.1186/s12889-022-13810-9

**Published:** 2022-07-22

**Authors:** Ogilvie Thom, Kym Roberts, Peter A. Leggat, Sue Devine, Amy E. Peden, Richard C. Franklin

**Affiliations:** 1 Department of Emergency Medicine, Sunshine Coast Hospital and Health Service, Birtinya, QLD Australia; 2 College of Public Health, Medical and Veterinary Sciences, James Cook University, Townsville, QLD Australia; 3 Surf Life Saving Queensland, Brisbane, QLD Australia; 4 School of Medicine, National University of Ireland Galway, Galway, Ireland; 5 Royal Life Saving Society – Australia, Sydney, NSW Australia; 6 School of Population Health, UNSW Sydney, Kensington, NSW Australia

**Keywords:** Cervical spine injury, Risk of drowning, Shallow water diving

## Abstract

**Objective:**

Surf zone injuries include cervical spine injuries (CSI). Risk factors for CSI have not been extensively investigated. The objective was to examine risk factors associated with diagnosed CSI that occurred in a beach setting.

**Methods:**

This retrospective case series used manually linked data from Sunshine Coast Hospital and Health Service Emergency Departments, Queensland Ambulance Service, Surf Life Saving Queensland (SLSQ), and Bureau of Meteorology data from 01/01/2015-21/04/2021. Variables included victim demographics, mechanism of injury, scene information, and patient course.

**Results:**

Seventy-nine of the 574 (13.8%) confirmed CSI occurred at the beach. Local residents and visitors were injured equally. Females represented a minority (12.7%) of those diagnosed with CSI but were a higher proportion of suspected spinal incidents reported to SLSQ (45%). Surfers were more likely to be injured through shallow water diving than swimmers (27.6% vs 2.2%). Females were more likely to be injured by shallow water diving than males (30.0% vs 8.7%). Visitors were more likely to be injured swimming and local residents surfing (68.2% vs 77.8% respectively). CSI occurred most commonly (40.0%) with a below average ocean wave height (0.75-1.25 m) and were most likely (45.3%) to occur in the second half of the outgoing tide. One beach had a statistically significant greater incidence of spinal incidents (OR 3.9, 95% CI: 2.1-7.2) and of CSI (OR 10.7, 95% CI: 1.5-79.5).

**Conclusions:**

Risk factors for CSI at the beach include male sex, smaller wave height and an outgoing tide. Shallow water diving among surfers and females should be addressed urgently.

**Supplementary Information:**

The online version contains supplementary material available at 10.1186/s12889-022-13810-9.

## Introduction

Australia is renowned for its beaches and beach culture with eleven million people visiting the coast to wade, swim or surf in 2020, of which 3.9 million people did so frequently [1]. Beaches can be hazardous environments with rips, waves, currents, rocks and sandbars causing risk of injury and death [2]. Hazardous surf conditions, when coinciding with peak beach visitation periods, increase the risk [3]. It has previously been shown that injuries caused by waves in the surf zones of beaches are common and often serious [4, 5]. Wave-forced impacts causing a head-first collision with the ocean floor can result in an axial load down the cervical spine, a very high-risk mechanism for cervical spine injury (CSI) [6]. Cervical spine injuries caused by wave-forced impact have been reported in swimmers and surfers in studies from the United States of America [5, 7], France [4], United Kingdom [8], Mexico [9, 10] and Australia [11].

The energy per square meter of a wave is proportional to the square of the height of the wave [12]. Thus, a two-metre wave will have four times the force of a one metre wave. However, when the influence of wave size on surf zone injuries was previously examined, the highest injury rates were noted in moderate (0.6 m) waves, with lower rates in larger and smaller waves [5]. A similar relationship has been reported between wave height and tide levels and lifeguard rescues [13]. Other reported risk factors for surf zone injuries include male sex [4, 7, 8, 11] and being a visitor [4, 7, 8]. However, risk factors for CSI occurring at the beach have not been extensively studied.

Beach hazard assessment typically combines the morphological characteristics of the beach, local features such headlands and reefs, and transient hazards such as breaking waves and rip currents [2]. The Australian Beach Safety and Management Program (ABSAMP) has complied a database of the physical characteristics and hazard ratings of 12,000 beach systems in Australia [2]. This information is available online at beachsafe.org.au [14]. While the risk assessment for a beach will include predictable environmental hazards, it must also include information regarding human factors, such as the characteristics and behaviours of beach users, information which is frequently difficult and expensive to obtain [2].

The aim of this study was to report on the epidemiology and risk factors for CSI occurring at beaches in the Sunshine Coast in Queensland, Australia for the purpose of informing future injury prevention strategies.

## Methods

This retrospective case series included all patients who presented to the three Emergency Departments (ED) of the Sunshine Coast Hospital and Health Service (SCHHS) with diagnosed CSI that occurred at a beach between 01/01/2015 and 21/04/2021. This study received ethical approval from The Prince Charles Hospital Human Research and Ethics Committee (Project no: 49754) and James Cook University Human Research Ethics Committee (H8014). All methods were carried out in accordance with relevant guidelines and regulations.

The Sunshine Coast is located in South-East Queensland, approximately 100 km north of Brisbane, Australia, and has many popular surf beaches. In 2020, it had a population of 393,039 [15]. The Sunshine Coast is a frequent destination for visitors with over 8.5 million visitor overnight stays and over 4.5 million visitor day visits during the period 1-July-2019 to 30-June-2020 [16]. Until 2017, it was served by the Emergency Departments of Nambour General Hospital and Caloundra Hospital. In 2017 the Sunshine Coast University Hospital opened and became the tertiary referral centre for the Sunshine Coast with the Emergency Department at Caloundra Hospital closing.

Data sources included SCHHS Integrated electronic Medical Record and ED Information System as well as the SLSQ Lifesaving Incident Management System and Operations Console (LIMSOC) electronic databases. Our search strategy is presented in supplementary file 1. Our search only identified patients with confirmed CSI and included patients with multiple diagnoses as long as they met the criteria of having a confirmed diagnosis of CSI. The search did not include patients initially suspected of having CSI and subsequently cleared of having the injury. Cervical spine injury was defined as bony or ligamentous injury to the cervical spine. The symptoms and signs of CSI are those typical of broken bones anywhere else in the body, such as pain and a limited range of motion. Cervical spine injuries can result in, but differ from, spinal cord injury, which may result in devastating neurological consequences, such as quadriplegia or death. Data from LIMSOC were only available from September 2016 onwards and included brief reports by SLSQ lifesavers and lifeguards of incidents such as rescues, resuscitations, potential spinal injuries and provision of first aid. Queensland Ambulance Service (QAS) case record forms were accessed as part of the medical record or obtained directly from QAS, when not included in the medical record.

The Sunshine Coast has 11 Surf Life Saving clubs, servicing 12 patrolled beaches. Eleven of the patrolled beaches have a general hazard rating of either 5 or 6 out of ten (moderately hazardous), while one has a hazard rating of 3 out of ten [14]. Interestingly, the beach described as the safest swimming beach on the Sunshine Coast has a hazard rating of 5/10 [14]. Beach visitor numbers were obtained from SLSQ. All SLSQ patrolled beaches routinely provide beach visitor estimates several times each patrolling day with many beaches patrolled 365 days a year by a combination of professional lifeguards and volunteer lifesavers. Both the professional lifeguards and volunteer lifesavers are trained and annually credentialled by SLSQ.

Tide and wave data for the study period were obtained from the Bureau of Meteorology (BOM) (www.bom.gov.au). Wave data for the region is recorded at 30-minute intervals from the Mooloolaba wave buoy, located 8 km offshore in approximately 30 metres of water depth. All confirmed CSI and SLSQ potential spinal injuries with geographic location and time data available (n=292) were used for the tide and wave height analysis.

Concerning the activity being undertaken, surfing was defined by the use of a rigid or finned board that would preclude its use in a swimming area patrolled by lifeguards for safety reasons. Use of body boards was classified as swimming. We chose this classification due to the fact that the most common cause of injuries to surfers is the board itself [11, 17, 18]. Locals were defined as having a residential postcode within one of the two Sunshine Coast local government areas (Sunshine Coast and Noosa). The tide was classified into either ebb or flood depending on whether the tide was outgoing (ebb) or incoming (flood) and by the number of hours from the preceding high or low tide.

Data were abstracted on a standardised case report form by two investigators (OT, KR) and entered into an Excel (Microsoft) spreadsheet. Variables recorded and the data sources (in hierarchical order) included victim demographics (such as age, sex, residential postcode, activity being undertaken and mechanism of injury) (EMR > QAS>LIMSOC), scene information including geographic location (QAS > LIMSOC > EMR), tide classification, wave height (BOM), beach visitor numbers and SLSQ suspected spinal incidents (LIMSOC) and beach hazard rating from the SLSQ beachsafe.com. au website [14].

Statistical analysis was conducted using IBM SPSS (version 27, Armonk, NY: IBM Corp.). Descriptive statistics were presented using median and inter-quartile range (IQR) when data were not normally distributed. Normality was assessed using the Shapiro-Wilk test [19]. A two-sided p value of less than 0.05 was considered statistically significant. Categorical variables were described using frequencies and percentages. We accounted for human interactions with the beach environment by converting the number of confirmed CSI and SLSQ suspected spinal incidents into ratios using the visitor numbers for each beach location.

## Results

There were 574 confirmed CSI who attended the ED during the study period. Seventy-nine (13.8%) were injured at the beach. There were 267 suspected spinal injuries documented by SLSQ on Sunshine Coast beaches with 30 (11.2%) being subsequently diagnosed with CSI in the ED.

Median age of the 79 CSI patients injured at the beach was 53 years (IQR 38-63). Patients experiencing a suspected spinal incident at the beach (147/267, 55.0%) and a diagnosed CSI were most likely to be male (69/79, 87.3%). There were 50,822,644 beach users recorded by SLSQ during the study period, giving a CSI rate of 1.6 per million beach users (79/50,822,644). The most common activities undertaken by patients with beach related CSI were swimming (54/79, 68.5%) and surfing (29/79, 36.7%). Sixty-six (83.5%) of the beach related CSI occurred in the warmer months between November and April, with no difference between locals and visitors (Table 1).

**Table 1:** Baseline characteristics of patients with cervical spine injuries

	Females	Males	Total
Number	10	69	79
Age (yrs) Median (IQR)	54 (27-65)	52 (39-63)	53 (38-63)
Activity *n*, (%)			
Swimming	4 (40.0)	40 (57.9)	44 (55.7)
Surfing	5 (50.0)	24 (34.7)	29 (36.7)
Other	1 (10.0)	1 (1.4)	2 (2.5)
Not recorded	0	4 (5.8)	4 (5.1)
Mechanism *n*, (%)			
Wave-forced impact	7 (70.0)	63 (91.3)	70 (88.6)
Shallow water diving	3 (30.0)	6 (8.7)	9 (11.4)
Local resident *n*, (%)			
Yes	4 (40.0)	35 (50.7)	39 (49.4)
No	4 (40.0)	34 (49.3)	38 (48.1)
Unknown	2 (20.0)	0	2 (2.5)

Seventy (88.6%) of the injuries were caused by wave-forced impact and 9 (11.4%) were the result of shallow water diving. All but one (8/9, 88.9%) of those injured by shallow water diving were surfing at the time of the injury. While there was no difference between locals and visitors with regard to the mechanism of injury, locals and visitors were injured undertaking different activities. Visitors were more likely to be injured swimming (30/44, 68.2%) and locals injured while surfing (21/27, 77.8%). Locals (10/39, 25.6%) were less likely to be attended by lifeguards/lifesavers than visitors (20/38, 52.6%). Swimmers were also more likely to present to SLSQ prior to the ED than surfers (24/44, 54.5% vs 5/29, 17.2%).

**Fig. 1:**
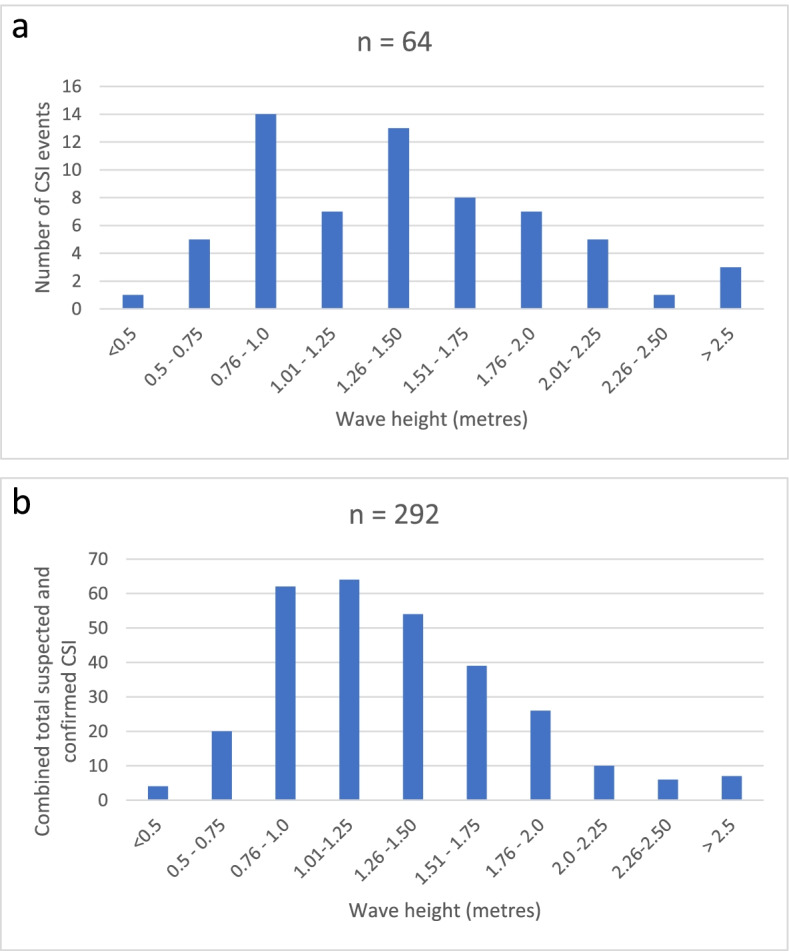
Graphical representation of wave height at time of CSI (**a**), *n*= 64 and combined suspected and confirmed spinal incidents (**b**) occurring at the beach

The mean wave height for the Sunshine Coast from January 2015 to April 2021 was 1.20 metres (m). The time and geographical location of the CSI were recorded in 64/79 (81.0%) of cases and 258/267 (96.7%) of the suspected spinal cases. The mean wave height at the time of occurrence of CSI was 1.40m (SD ± 0.59m). However, as shown in Fig. 1, the single highest frequency (14/64, 21.9%) of CSI occurred when wave height was below average (0.76-1.0 m). The results were similar when all spinal incidents were analyzed, with 62/292 (21.2%) and 64/292 (21.9%) injured when the waves were between 0.76-1.0 m and 1.01-1.25 m, respectively. There was no relationship between ocean wave height and activity (surfing and swimming) or visitor status.

**Fig. 2:**
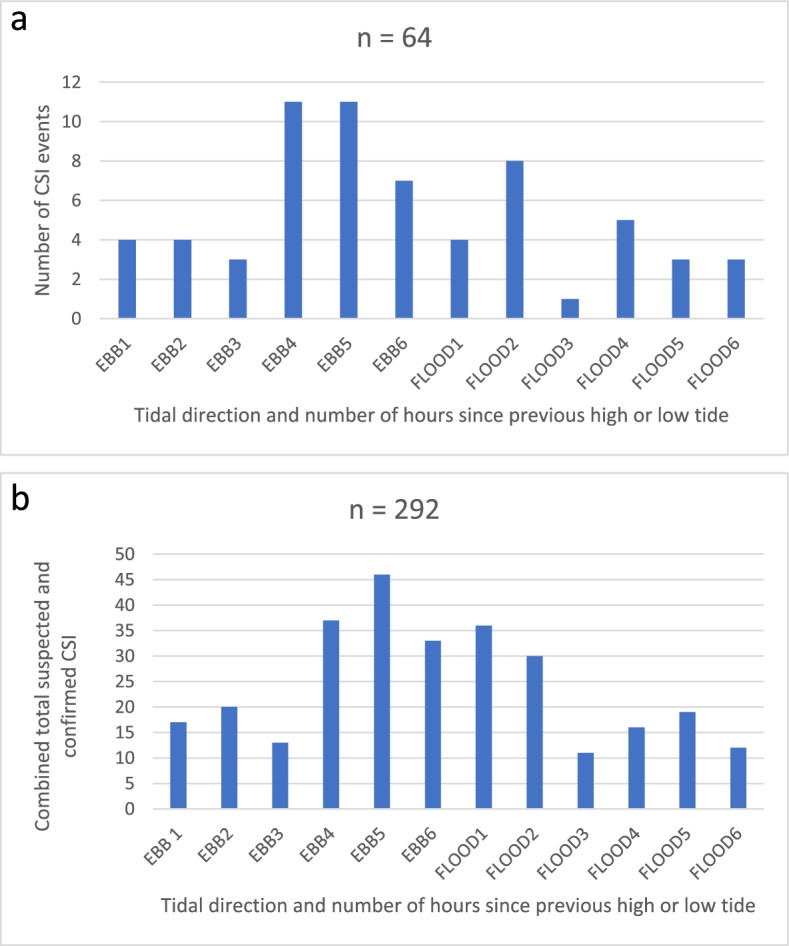
Tide level at time of CSI (**a**) and combined suspected and confirmed spinal injuries (**b**) occurring at the beach

The relationship between CSI and tide is represented graphically in Fig. 2. Forty-five percent (29/64) of the injuries occurred in the last half of the ebb (outgoing) tide, while the first half of the flood tide had 13 (20%) of the CSI. The combined analysis of all spinal incidents had very similar results with 116/292 (39.7%) for the last half of the ebb tide and 77/292 (26.3%) for the first half of the flood tide. The higher tides, the second half of the flood tide and the first half of the ebb tide were associated with 11 (17%) of the CSI and 97/292 (33.2%) of the combined analysis. There was no relationship between tide level and activity, visitor status or beach location.

The ratios per beach visitor of potential and confirmed CSI for each location is presented in Table 2, along with calculated relative risk. Alexandra Headland was used as the reference for both suspected spinal incidents and confirmed CSI. There was a single location (Mooloolaba Beach) that had a relative risk significantly higher for both CSI (RR 10.7, 95% CI 1.5-79.5) and suspected spinal incidents (RR 3.9, 95% CI 2.1-7.2) than other locations.

**Table 2:** – Relative risk of CSI and spinal incident by beach location

Beach Location	Beach Safety Rating (SLSQ)	CSI	SLSQ Spinal Incidents	No: of beach users (BU)	Rate of CSI per million BU	Rate spinal Incidents per million BU	RR (95% CI) CSI C/W Alexandra Headland	RR (95% CI) spinal incident C/W Alexandra Headland
Alexandra Headland	5/10	1	11	5,850,667	0.17092	1.88013	-	
Buddina	6/10	1	1	856,941	1.16694	1.16694	6.8 (0.4-109.0)	0.6 (0.1-4.8)
Currimundi	6/10	1	3	1,498,160	0.66749	2.00246	3.9 (0.2-62.4)	1.1 (0.3-3.8)
Marcoola	6/10	1	5	751,148	1.3313	6.65648	7.8 (0.5-124.5)	3.5 (1.2-10.2)
Maroochydore	5/10	1	11	3,850,021	0.25974	2.85713	1.5 (0.1-24.3)	1.5 (0.7-3.5)
Peregian	6/10	1	6	1,351,553	0.73989	4.43934	4.3 (0.3-69.2)	2.3 (0.9-6.4)
Rainbow	5/10	1	9	1,571,802	0.63621	5.72591	3.7 (0.2-59.5)	3.0 (1.3-7.3)
Sunrise	6/10	1	-	477,644	2.09361	-	12.2 (0.8-195.8)	-
Coolum	6/10	2	18	4,369,325	0.45774	4.11963	2.7 (0.2-29.5)	2.2 (1.0-4.6)
Dicky	5/10	2	9	2,294,214	0.87176	3.92291	5.1 (0.5-56.3)	2.1 (0.9-5.0)
Sunshine	6/10	2	6	2,076,501	0.96316	0.288948	5.6 (0.5-62.1)	1.5 (0.6-4.2)
Mudjimba	6/10	3	6	1,547,876	1.93814	3.87628	11.3 (1.2-109.0)	2.1 (0.8-5.6)
Caloundra	5/10	4	28	3,698,370	1.08156	7.5709	6.3 (0.7-56.6)	4.0 (2.0-8.1)
Noosa	3/10	11	60	14,534,367	0.75683	4.12815	4.4 (0.6-34.3)	2.2 (1.2-4.2)
Mooloolaba	5/10	22	87	12,006,163	1.83239	7.24628	10.7 (1.5-79.5)	3.9 (2.1-7.2)
Totals		54	260	50,822,644				

## Discussion

Aside from drowning, CSI represents one of the most devastating consequence of a visit to the beach. Fortunately, they are uncommon, occurring at a rate of 1-2 per million beach user. However, with millions of people visiting the beach every year, they occur frequently enough to represent 14% of all the CSI over the six plus year period of this study. This study found differences in the mechanism of injury between males and females, as well as between swimmers and surfers. We also demonstrated that smaller waves and an ebb tide are environmental risk factors for CSI occurring at the beach.

The typical person injured in our study was a middle aged (53 years old) male. While this is more than ten years older than similar studies from the United States [7, 20], the predominance of male patients in this study, with only 12.9% being female, is similar to multiple studies examining surf zone injuries where females represented between 0 and 30% of those injured [4, 5, 7–9]. However, the proportion of females with suspected spinal injuries reported to SLSQ was much higher at 45%. The reason for this is not clear with inconsistent differences between sexes noted in risk taking behaviours at the beach [21] and exposure with males more likely to go to the beach to surf than females [22]. However, in this study, females were more likely than males to sustain a CSI while surfing (5/10, 50% vs 24/69, 35%) at the beach than swimming (4/9, 44% vs 40/69, 58%). There may be local differences in beach culture that account for these findings or the popularity of surfing among females may have increased.

Eight of the nine CSI sustained by shallow water diving were surfers. Being dumped by waves and ‘wiped out’ is an integral part of surfing and a common cause of injuries [18]. Although there were no CSI reported in the study by Taylor [18], others have reported CSI in surfers, albeit at low frequencies [8]. Our study found a four to one ratio of wave-forced impacts vs shallow water diving across all participants, which is similar to results from Hawai’i [7], however the activities being undertaken at the time of injury were not reported in Hawai’i. A recent study in Australian surfers found that the majority of surfers injured were experienced [11]. Further study into shallow water diving by surfers would allow focused preventive measures to be developed and implemented. For example, if they are largely novice surfers, an educational campaign advising a feet first dismount from the board at all times should be aimed at the numerous surf schools that operate in the region. A similar campaign should be aimed at the various board-riding clubs and associations, if it appears more experienced surf-board riders are being injured this way.

We found that local residents and visitors were split evenly amongst our patients. This differs from previous reports where visitors are significantly more likely to suffer CSI than locals [7, 9] or to be injured in surf zones [4, 5]. We did discover differences in behaviour between locals and visitors with visitors more likely to be injured swimming rather than surfing and to be attended by lifeguards. It seems logical that visitors were injured swimming rather than surfing – a surfboard is more difficult to transport long distances than swimwear. Also, given that visitors are less likely to be familiar with safe swimming locations and thus swim between the flags, the increased proportion of visitors presenting to lifeguards seems reasonable. Another explanation is that visitors would seek out lifeguards post injury as the first link in the chain to receiving medical attention, if they were unfamiliar with the location of the Sunshine Coast hospitals. Locals may also have beach access away from the main centres of tourist accommodation where the beaches are less likely to be patrolled and be familiar with the less crowded surf breaks away from popular beaches.

We used beach user recorded by SLSQ as proxy for exposure to risk. We believe that locals may wait for favourable conditions on the water or good-sized waves before swimming or surfing. Visitors to the area may swim or surf in conditions that locals will not, simply because of a lack of other opportunity. The differences in behaviours between locals and visitors is clearly an area that needs further exploration.

The difference in mean wave height associated with CSI compared to the mean wave height for the Sunshine Coast (1.40 m vs 1.20 m) was not unexpected. However, the largest single frequency of CSI (14/91, 15%) occurred when the wave height was well below average (0.75-1.0 m). This supports similar findings from the US where the highest rates for being injured in surf zone were seen in days associated with small waves (0.6 m) and that days of larger waves were associated with fewer injuries [5]. This deterrent effect of large waves has also been reported in relation to the number of lifeguard rescues being performed [13]. We postulate that the injuries occurring with the smaller waves occur in the more inexperienced swimmers and surfers, who were encouraged into the water by the safer appearing waves. However, our study found no obvious relationship between wave height and visitor status as a de-facto indicator of experience level. Another explanation may lie in the smaller waves breaking in shallower water, thereby increasing the likelihood of collision with the ocean floor [7]. The increased frequency of CSI in smaller waves could also be a reflection of increased numbers of water users with the safer appearing waves. Unfortunately, we don’t have that data available. Future research should explore this as well as the experience and skill levels of swimmers and surfers with CSI.

CSI were more likely to occur on the last half of the outgoing, or ebb, tide. We postulated that this might be more likely in surfers than in swimmers, as swimmers are frequently in contact with the ocean floor, and consequently have an awareness of the depth of the ocean at their location. However, there was no statistical difference between swimmers and surfers. Another potential explanation is the increase in wave size associated with the ebb tide.

**Fig. 3:**
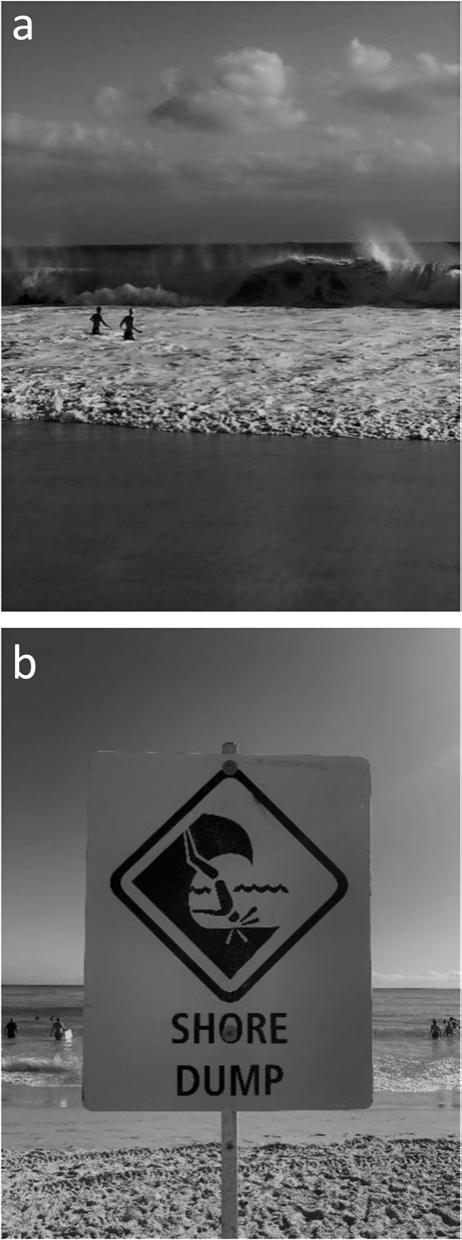
**a** Hazardous shore break at Mooloolaba Beach, **b** Warning sign at Mooloolaba Beach

Finally, our study identified one beach in particular as having a statistically significant higher rate of both spinal incidents presenting to lifeguards and CSI diagnosed in the ED compared with other locations. This is concordant with the findings of Chang, where different beaches have a differing risk profile with the majority of wave related CSI occurring on beaches with a severe shore-break, high energy waves with plunging characteristics, breaking on a steep sloped ocean floor (Fig. 3a) [7]. The dangerous nature of the shore-break at Mooloolaba is well recognised with warning signs in place (Fig. 3b). However, this beach is very popular with over 12 million recorded beach users during the study period and it is described as the safest swimming beach on the Sunshine Coast [14], though it is given a ABSAMP hazard rating of 5/10 (moderately dangerous). However, even blatantly dangerous conditions do not prevent locals and visitors alike from entering the water (Fig. 3a). Clearly, risk assessment, environment (including access) and behavioural choices at the beach need to be studied further. Complicating this issue is the fact that the highest frequency of injuries occur in conditions with small waves, when beaches are open for swimming.

### Strengths and limitations

Our study is the second largest patient series globally to examine risk factors for CSI at the beach and the first to examine the influence of wave size and tide on the occurrence of CSI. The beaches of the Sunshine Coast stretch for approximately 60 km of coastline so data from a single wave buoy (located approximately halfway along the coast) may not be truly representative for each location. Secondly, the relationship between ocean wave height and the height and characteristics of waves in the surf zone is complex with shifting sand banks, rip currents, tidal currents and the wind all influencing the behaviour of waves in the surf zone. We were unable to access wave height predictions/measurements for each beach location for the duration of the study and hence relied on the wave buoy data.

As mentioned earlier, our search strategy only identified patients with confirmed CSI, not the patients investigated for CSI but cleared of any injury. Given the comprehensive nature of the strategy (supplementary file 1), we are confident the number of missed patients with CSI is minimal. The suspected spinal injuries reported on the SLSQ LIMSOC database that could not be data linked occurred on beaches in close proximity to our study hospitals. The nearest other Emergency Department and trauma centre are 50 and 100 km away, respectively. Given the geographic distance to other hospitals and our comprehensive search strategy for case identification, we believe that the patients with suspected spinal injuries from the SLSQ database that could not be data linked most likely attended SCHHS but were subsequently cleared of having CSI.

Future research should examine the skill and experience levels of the swimmers and surfers involved, especially concerning the injuries occurring in smaller, but perhaps not safer, waves. Beach numbers have been used as a proxy for exposure and may not accurately reflect exposure, as it has been postulated that visitors will enter more hazardous surf whereas local will wait for better conditions. These figures are collected by lifeguards and lifesavers with no formal training in counting populations. As such, the accuracy of these figures cannot be ascertained. However, these figures have been collected several times a day, every day of the year, on patrolled beaches and are currently the best estimation of beach usage available.

## Conclusions

Our study demonstrates that the person most likely to sustain a cervical spine injury at the beach is a middle-aged male. However, we also found an unexpectedly high number of surfers and females injured by diving into shallow water. This must be addressed urgently with preventative measures. Swimmers and surfers were most likely injured on smaller than average waves and during the last half of the outgoing tide. A single hotspot location for both suspected spinal injuries and confirmed CSI was identified. Locals and visitors were injured equally, although they were injured doing different things. These findings should inform future injury prevention campaigns.

## Supplementary Information

**Additional file 1.**

## Data Availability

The datasets generated and/or analysed during the current study are not publicly available due to the Public Health Act 2005, Queensland. Application can be made to Prince Charles Hospital HREC for access.
