# Supplementary material for: Cervical spine injuries occurring at the beach: epidemiology, mechanism of injury and risk factors
Source: BMC Public Health. 2022 Jul 22;22:1404. doi: 10.1186/s12889-022-13810-9 (PMC9306180; doi:10.1186/s12889-022-13810-9)
Supplement: Supplementary file 1 — Additional file 1. [file 12889_2022_13810_MOESM1_ESM.docx]

**SEARCH 1. SNOMED Drowning and immersion categories**

[Accident caused by immersion, suffocation, foreign body (event)](https://snomedbrowser.com/Codes/Details/217747005)

[Accident to watercraft causing immersion (event)](https://snomedbrowser.com/Codes/Details/215415000)

[Accidental drowning and submersion](https://snomedbrowser.com/Codes/Details/217748000)

[Accidental drowning and submersion in swimming pool](https://snomedbrowser.com/Codes/Details/217776008)

[Accidental drowning and submersion while hunting, except from boat or with diving equipment (event)](https://snomedbrowser.com/Codes/Details/217757006)

[Accidental drowning and submersion whilst engaged in sport or recreational activity without diving equipment](https://snomedbrowser.com/Codes/Details/217755003)

[Accidental drowning and submersion whilst engaged in sport or recreational activity with diving equipment](https://snomedbrowser.com/Codes/Details/217750008)

[Accidental drowning or immersion during marine salvage (event)](https://snomedbrowser.com/Codes/Details/217766005)

[Accidental drowning or immersion during placement fishing nets (event)](https://snomedbrowser.com/Codes/Details/217768006)

[Accidental drowning or immersion during recreation](https://snomedbrowser.com/Codes/Details/242661008)

[Accidental drowning or immersion during rescue attempt person](https://snomedbrowser.com/Codes/Details/217769003)

[Accidental drowning or immersion during underwater construction (event)](https://snomedbrowser.com/Codes/Details/217770002)

[Accidental drowning or immersion during underwater repairs](https://snomedbrowser.com/Codes/Details/217771003)

[Accidental drowning or immersion in bathtub](https://snomedbrowser.com/Codes/Details/217773000)

[Accidental drowning or immersion in course of work (event)](https://snomedbrowser.com/Codes/Details/242660009)

[Accidental drowning or immersion in quenching tank (event)](https://snomedbrowser.com/Codes/Details/217775007)

[Accidental drowning or immersion while fishing from shore, bank or fixed structure (event)](https://snomedbrowser.com/Codes/Details/217756002)

[Accidental drowning or immersion while ice skating (event)](https://snomedbrowser.com/Codes/Details/217758001)

[Accidental drowning or immersion while playing in water (event)](https://snomedbrowser.com/Codes/Details/217759009)

[Accidental drowning or immersion while wading in water (event)](https://snomedbrowser.com/Codes/Details/217762007)

[Accidental drowning or immersion while water skiing (event)](https://snomedbrowser.com/Codes/Details/217749008)

[Accidental drowning or immersion whilst pearl diving](https://snomedbrowser.com/Codes/Details/217767001)

[Accidental drowning or immersion whilst surfboarding](https://snomedbrowser.com/Codes/Details/217761000)

[Accidental drowning or immersion whilst swimming](https://snomedbrowser.com/Codes/Details/242662001)

[Accidental drowning or immersion whilst windsurfing](https://snomedbrowser.com/Codes/Details/242663006)

[Accidental drowning or near drowning whilst scuba diving](https://snomedbrowser.com/Codes/Details/242664000)

[Accidental drowning or near drowning whilst skin diving](https://snomedbrowser.com/Codes/Details/242665004)

[Accidentally drowned by machinery](https://snomedbrowser.com/Codes/Details/217921005)

[Aspiration pneumonia due to near drowning (disorder)](https://snomedbrowser.com/Codes/Details/72854003)

[Assault by drowning](https://snomedbrowser.com/Codes/Details/219197006)

[Attempted suicide - drowning (event)](https://snomedbrowser.com/Codes/Details/287183002)

Cardiac arrest due to drowning

[Decomposed body with immersion](https://snomedbrowser.com/Codes/Details/373384007)

[Decomposed body without immersion (finding)](https://snomedbrowser.com/Codes/Details/371644000)

Drowning

[Drowning and non-fatal immersion](https://snomedbrowser.com/Codes/Details/212962007)

[Drowning in brackish water (event)](https://snomedbrowser.com/Codes/Details/7420003)

[Drowning in fresh water](https://snomedbrowser.com/Codes/Details/50109008)

[Drowning in liquid other than water](https://snomedbrowser.com/Codes/Details/15654006)

[Drowning in saltwater](https://snomedbrowser.com/Codes/Details/74376000)

[Drowning or immersion of unknown intent (event)](https://snomedbrowser.com/Codes/Details/219333004)

[Drowning other person (finding)](https://snomedbrowser.com/Codes/Details/284644007)

[Drowning self (finding)](https://snomedbrowser.com/Codes/Details/225050000)

[Dry drowning](https://snomedbrowser.com/Codes/Details/242014006)

[Immersion](https://snomedbrowser.com/Codes/Details/19674006)

[Immersion hypothermia (disorder)](https://snomedbrowser.com/Codes/Details/241968001)

[Immersion or drowning due to being washed overboard (event)](https://snomedbrowser.com/Codes/Details/271582003)

[Immersion or drowning due to fall from gangplank](https://snomedbrowser.com/Codes/Details/215581001)

[Immersion or drowning due to fall overboard from vessel without accident to vessel (event)](https://snomedbrowser.com/Codes/Details/242181005)

[Immersion or drowning due to falling from vessel in collision with fixed structure](https://snomedbrowser.com/Codes/Details/242174003)

[Immersion or drowning due to falling from vessel into water (event)](https://snomedbrowser.com/Codes/Details/242172004)

[Immersion or drowning due to falling from vessel on fire](https://snomedbrowser.com/Codes/Details/269667001)

[Immersion or drowning due to vessel sinking (event)](https://snomedbrowser.com/Codes/Details/269666005)

[Submersion and drowning due to boat overturning](https://snomedbrowser.com/Codes/Details/269665009)

[Submersion and drowning due to boat submerging (event)](https://snomedbrowser.com/Codes/Details/215427002)

[Submersion and drowning due to falling from burning ship](https://snomedbrowser.com/Codes/Details/215437007)

[Submersion and drowning due to falling from crushed watercraft (event)](https://snomedbrowser.com/Codes/Details/215458001)

[Submersion and drowning due to jumping from burning ship (event)](https://snomedbrowser.com/Codes/Details/215447005)

[Submersion and drowning due to jumping from crushed watercraft](https://snomedbrowser.com/Codes/Details/215468006)

[Submersion or drowning due to being thrown overboard (event)](https://snomedbrowser.com/Codes/Details/215601006)

[Submersion or drowning due to being thrown overboard, docker or stevedore injured](https://snomedbrowser.com/Codes/Details/215608000)

[Submersion or drowning due to being thrown overboard, occupant of small powered boat injured (event)](https://snomedbrowser.com/Codes/Details/215603009)

[Submersion or drowning due to being thrown overboard, occupant of small unpowered boat injured](https://snomedbrowser.com/Codes/Details/215602004)

[Submersion or drowning due to being thrown overboard, swimmer injured (event)](https://snomedbrowser.com/Codes/Details/215607005)

[Submersion or drowning due to being thrown overboard, water skier injured](https://snomedbrowser.com/Codes/Details/215606001)

[Submersion or drowning due to being washed overboard, docker or stevedore injured](https://snomedbrowser.com/Codes/Details/215619002)

[Submersion or drowning due to being washed overboard, occupant of small powered boat occupant](https://snomedbrowser.com/Codes/Details/215613001)

[Submersion or drowning due to being washed overboard, occupant of small unpowered boat injured](https://snomedbrowser.com/Codes/Details/215612006)

[Submersion or drowning due to being washed overboard, swimmer injured (event)](https://snomedbrowser.com/Codes/Details/215618005)

[Submersion or drowning due to being washed overboard, water skier injured (event)](https://snomedbrowser.com/Codes/Details/215616009)

[Submersion or drowning due to boat overturning, docker or stevedore injured](https://snomedbrowser.com/Codes/Details/215424009)

[Submersion or drowning due to boat overturning, occupant of small powered boat injured (event)](https://snomedbrowser.com/Codes/Details/215418003)

[Submersion or drowning due to boat overturning, occupant of small unpowered boat injured (event)](https://snomedbrowser.com/Codes/Details/215417008)

[Submersion or drowning due to boat overturning, swimmer injured (event)](https://snomedbrowser.com/Codes/Details/215423003)

[Submersion or drowning due to boat overturning, water skier injured (event)](https://snomedbrowser.com/Codes/Details/215422008)

[Submersion or drowning due to boat submerging, docker or stevedore injured](https://snomedbrowser.com/Codes/Details/215434000)

[Submersion or drowning due to boat submerging, occupant of small powered boat injured (event)](https://snomedbrowser.com/Codes/Details/215429004)

[Submersion or drowning due to boat submerging, occupant of small unpowered boat injured (event)](https://snomedbrowser.com/Codes/Details/215428007)

[Submersion or drowning due to boat submerging, swimmer injured](https://snomedbrowser.com/Codes/Details/215433006)

[Submersion or drowning due to boat submerging, water skier injured](https://snomedbrowser.com/Codes/Details/215432001)

[Submersion or drowning due to fall from gangplank, docker or stevedore injured](https://snomedbrowser.com/Codes/Details/215588007)

[Submersion or drowning due to fall from gangplank, occupant of small powered boat injured (event)](https://snomedbrowser.com/Codes/Details/215583003)

[Submersion or drowning due to fall from gangplank, occupant of small unpowered boat injured (event)](https://snomedbrowser.com/Codes/Details/215582008)

[Submersion or drowning due to fall from gangplank, swimmer injured](https://snomedbrowser.com/Codes/Details/215587002)

[Submersion or drowning due to fall from gangplank, water skier injured (event)](https://snomedbrowser.com/Codes/Details/215586006)

[Submersion or drowning due to fall overboard (event)](https://snomedbrowser.com/Codes/Details/215591007)

[Submersion or drowning due to fall overboard, docker or stevedore injured (event)](https://snomedbrowser.com/Codes/Details/215598001)

[Submersion or drowning due to fall overboard, occupant of small powered boat injured](https://snomedbrowser.com/Codes/Details/215593005)

[Submersion or drowning due to fall overboard, occupant of small unpowered boat injured](https://snomedbrowser.com/Codes/Details/215592000)

[Submersion or drowning due to fall overboard, swimmer injured](https://snomedbrowser.com/Codes/Details/215597006)

[Submersion or drowning due to fall overboard, water skier injured (event)](https://snomedbrowser.com/Codes/Details/215596002)

[Submersion or drowning due to falling from burning ship, docker or stevedore injured](https://snomedbrowser.com/Codes/Details/215444003)

[Submersion or drowning due to falling from burning ship, occupant of small powered boat injured](https://snomedbrowser.com/Codes/Details/215439005)

[Submersion or drowning due to falling from burning ship, occupant of small unpowered boat injured (event)](https://snomedbrowser.com/Codes/Details/215438002)

[Submersion or drowning due to falling from burning ship, swimmer injured](https://snomedbrowser.com/Codes/Details/215443009)

[Submersion or drowning due to falling from burning ship, water skier injured](https://snomedbrowser.com/Codes/Details/215442004)

[Submersion or drowning due to falling from crushed watercraft, docker or stevedore injured (event)](https://snomedbrowser.com/Codes/Details/215465009)

[Submersion or drowning due to falling from crushed watercraft, occupant of small powered boat injured](https://snomedbrowser.com/Codes/Details/215460004)

[Submersion or drowning due to falling from crushed watercraft, occupant of small unpowered boat injured (event)](https://snomedbrowser.com/Codes/Details/215459009)

[Submersion or drowning due to falling from crushed watercraft, swimmer injured](https://snomedbrowser.com/Codes/Details/215464008)

[Submersion or drowning due to falling from crushed watercraft, water skier injured](https://snomedbrowser.com/Codes/Details/215463002)

[Submersion or drowning due to jumping from burning ship, docker or stevedore injured (event)](https://snomedbrowser.com/Codes/Details/215455003)

[Submersion or drowning due to jumping from burning ship, occupant of small powered boat injured (event)](https://snomedbrowser.com/Codes/Details/215449008)

[Submersion or drowning due to jumping from burning ship, occupant of small unpowered boat injured](https://snomedbrowser.com/Codes/Details/215448000)

[Submersion or drowning due to jumping from burning ship, swimmer injured (event)](https://snomedbrowser.com/Codes/Details/215454004)

[Submersion or drowning due to jumping from burning ship, water skier injured (event)](https://snomedbrowser.com/Codes/Details/215453005)

[Submersion or drowning due to jumping from crushed watercraft, docker or stevedore injured (event)](https://snomedbrowser.com/Codes/Details/215475007)

[Submersion or drowning due to jumping from crushed watercraft, occupant of small powered boat injured](https://snomedbrowser.com/Codes/Details/215470002)

[Submersion or drowning due to jumping from crushed watercraft, occupant of small unpowered boat injured](https://snomedbrowser.com/Codes/Details/215469003)

[Submersion or drowning due to jumping from crushed watercraft, swimmer injured](https://snomedbrowser.com/Codes/Details/215474006)

[Submersion or drowning due to jumping from crushed watercraft, water skier injured](https://snomedbrowser.com/Codes/Details/215473000)

[Submersion or drowning due to ship sinking, docker or stevedore injured (event)](https://snomedbrowser.com/Codes/Details/215485008)

[Submersion or drowning due to ship sinking, occupant of small powered boat injured (event)](https://snomedbrowser.com/Codes/Details/215480003)

[Submersion or drowning due to ship sinking, occupant of small unpowered boat injured](https://snomedbrowser.com/Codes/Details/215479001)

[Submersion or drowning due to ship sinking, water skier injured](https://snomedbrowser.com/Codes/Details/215483001)

[Suicide - drowning](https://snomedbrowser.com/Codes/Details/287192004)

[Suicide and self-inflicted injury by drowning](https://snomedbrowser.com/Codes/Details/219141008)

[Suicide or attempted suicide by drowning](https://snomedbrowser.com/Codes/Details/269728002)

[Vasovagal syncope due to immersion (disorder)](https://snomedbrowser.com/Codes/Details/234166002)

[War injury, drowned in war operations](https://snomedbrowser.com/Codes/Details/219418008)

DROWNING KEYWORD search terms

LOCATION

Sea, ocean, surf, river, lake, pond, dam, pool, spa, bath, beach, shore, bridge, wave, waves, rip, current, water, fresh water, freshwater, saltwater, underwater, under water,

EVENT

Immersion, submersion, submerged, drowning, near-drowning, arrest, cardiac arrest, respiratory arrest, CPR, resuscitation, resus, dumped, dunked, tumbled, struggling, struggled, struggle, rescue, float, floating, lifeguard, life guard, life saver, lifesaver

ACTIVITY

Swim, swimming, swam, swimmer, surfer, paddler, canoe, kayak, surf ski, jet ski, boat, surfing, surfboard, capsize, capsizing, fishing,

**SEARCH 2. SNOMED Cervical spine categories**

Active range of cervical spine extension

Active range of cervical spine extension – finding

Active range of cervical spine flexion

Active range of cervical spine flexion - finding

Active range of cervical spine left lateral flexion

Active range of cervical spine left lateral flexion - finding

Active range of cervical spine left rotation

Active range of cervical spine left rotation - finding

Active range of cervical spine left side flexion

Active range of cervical spine protraction - finding

Active range of cervical spine retraction - finding

Active range of cervical spine right lateral flexion

Active range of cervical spine right lateral flexion - finding

Active range of cervical spine right rotation - finding

Active range of cervical spine right side flexion

Cervical spine – range of movement – finding

Cervical spine crepitus

Cervical spine crepitus audible

Cervical spine crepitus palpable

Cervical spine deformity

Cervical spine instability

Cervical spine range of motion

Closed dislocation cervical spine

Closed fracture of cervical spine

Closed fracture of cervical spine with cord lesion

Closed subluxation cervical spine

Compression fracture of cervical spine

Decreased active range cervical spine left lateral flexion

Decreased active range cervical spine right lateral flexion

Decreased active range of cervical spine extension

Decreased active range of cervical spine flexion

Decreased active range of cervical spine left lateral flexion

Decreased active range of cervical spine left rotation

Decreased active range of cervical spine left side flexion

Decreased active range of cervical spine protraction

Decreased active range of cervical spine retraction

Decreased active range of cervical spine right lateral flexion

Decreased active range of cervical spine right rotation

Decreased active range of cervical spine right side flexion

Decreased passive range cervical spine left lateral flexion

Decreased passive range cervical spine right lateral flexion

Decreased passive range of cervical spine extension

Decreased passive range of cervical spine flexion

Decreased passive range of cervical spine left lateral flexion

Decreased passive range of cervical spine left rotation

Decreased passive range of cervical spine left side flexion

Decreased passive range of cervical spine protraction

Decreased passive range of cervical spine retraction

Decreased passive range of cervical spine right lateral flexion

Decreased passive range of cervical spine right rotation

Decreased passive range of cervical spine right side flexion

Decreased range of cervical spine extension

Decreased range of cervical spine flexion

Decreased range of cervical spine flexion and extension

Decreased range of cervical spine movement

Finding of active range of cervical spine extension

Finding of active range of cervical spine flexion

Finding of active range of cervical spine left lateral flexion

Finding of active range of cervical spine left rotation

Finding of active range of cervical spine protraction

Finding of active range of cervical spine retraction

Finding of active range of cervical spine right lateral flexion

Finding of active range of cervical spine right rotation

Finding of cervical spine

Finding of passive range of cervical spine extension

Finding of passive range of cervical spine flexion

Finding of passive range of cervical spine left lateral flexion

Finding of passive range of cervical spine left rotation

Finding of passive range of cervical spine protraction

Finding of passive range of cervical spine retraction

Finding of passive range of cervical spine right lateral flexion

Finding of passive range of cervical spine right rotation

Finding of range of cervical spine extension

Finding of range of cervical spine flexion

Finding of range of cervical spine left lateral flexion

Finding of range of cervical spine left rotation

Finding of range of cervical spine protraction

Finding of range of cervical spine retraction

Finding of range of cervical spine right lateral flexion

Finding of range of cervical spine right rotation

Finding of range of extension of cervical spine

Finding of range of flexion of cervical spine

Fracture dislocation of cervical spine

Fracture of cervical spine

Fracture of cervical spine – no cord lesion

Fracture of cervical spine with cord lesion

Injury of cervical spine

Injury to ligament of cervical spine

Multiple fractures of cervical spine

No active range of cervical spine extension

No active range of cervical spine flexion

No active range of cervical spine left lateral flexion

No active range of cervical spine left rotation

No active range of cervical spine protraction

No active range of cervical spine retraction

No active range of cervical spine right lateral flexion

No active range of cervical spine right rotation

No cervical spine movement

No passive range of cervical spine extension

No passive range of cervical spine flexion

No passive range of cervical spine left lateral flexion

No passive range of cervical spine left rotation

No passive range of cervical spine protraction

No passive range of cervical spine retraction

No passive range of cervical spine right lateral flexion

No passive range of cervical spine right rotation

O/E – cervical spine abnormal

On examination – cervical spine abnormal

Open dislocation cervical spine

Open fracture of cervical spine

Open fracture of cervical spine with cord lesion

Pain in cervical spine

Passive range of cervical spine extension

Passive range of cervical spine extension – finding

Passive range of cervical spine flexion

Passive range of cervical spine flexion - finding

Passive range of cervical spine left lateral flexion

Passive range of cervical spine left lateral flexion - finding

Passive range of cervical spine left rotation

Passive range of cervical spine left rotation - finding

Passive range of cervical spine left side flexion

Passive range of cervical spine protraction - finding

Passive range of cervical spine retraction - finding

Passive range of cervical spine right lateral flexion

Passive range of cervical spine right lateral flexion - finding

Passive range of cervical spine right rotation - finding

Passive range of cervical spine right side flexion

Range of cervical spine left lateral flexion

Range of cervical spine left lateral flexion - finding

Range of cervical spine left rotation

Range of cervical spine left rotation - finding

Range of cervical spine left side flexion

Range of cervical spine protraction - finding

Range of cervical spine retraction - finding

Range of cervical spine right lateral flexion

Range of cervical spine right lateral flexion - finding

Range of cervical spine right rotation - finding

Range of cervical spine right side flexion

Range of extension of cervical spine

Range of extension of cervical spine – finding

Range of flexion of cervical spine

Range of flexion of cervical spine – finding

Rotational deformity of cervical spine

Subluxation of joint of cervical spine

Keyword Search Terms

Drowning

LOCATION

Sea, ocean, surf, river, lake, pond, dam, pool, spa, bath, beach, shore, bridge, wave, waves, rip, current, water, fresh water, freshwater, salt water, underwater, under water,

EVENT

Immersion, submersion, submerged, drowning, near-drowning, dumped, dunked, tumbled, struggling, struggled, struggle, rescue, float, floating, lifeguard, life guard, life saver, lifesaver

ACTIVITY

Swim, swimming, swam, swimmer, surfer, paddler, canoe, kayak, surf ski, jet ski, boat, surfing, surfboard, capsize, capsizing, fishing,

Cervical spine injury/subluxation

LOCATION

Surf, wave, pool, swimming pool water, water park, sand, sandbank, creek,

EVENT

Dumped, dive, diving, dove, axial injury, neck injury, neck strike, immersion

ACTIVITY

Surfboard, surfing, body board
